# Tobacco exposure in children and adolescents with chronic kidney disease: parental behavior and knowledge. A study from the Midwest Pediatric Nephrology Consortium 

**DOI:** 10.5414/CN108024

**Published:** 2014-04-24

**Authors:** Abiodun Omoloja, Adrienne Stolfi, Deepa Chand, Benjamin Laskin, Debbie Gipson, Hiren Patel, Jane Anne Smith, Aftab Chishti

**Affiliations:** 1 Wright State University, Dayton, OH,; 2 ush Children’s Hospital, Chicago, IL,; 3 The Children’s Hospital of Philadelphia, Division of Nephrology, Philadelphia, PA,; 4 University of Michigan, School of Medicine, Ann Arbor, MI,; 5 Nationwide Children’s Hospital, Division of Nephrology, Columbus, OH, and; 6 University of Kentucky Children’s Hospital, Division of Nephrology, Hypertension and Renal Transplantation, Lexington, KY, USA

**Keywords:** tobacco, pediatrics, chronic kidney disease

## Abstract

Aim: The incidence of cardiovascular disease (CVD) in children with chronic kidney disease (CKD) is high. Exposure to second hand smoke (SHS) is a known risk factor for CVD. Due to a recent report of high incidence of SHS in children with CKD, we sought to investigate via questionnaire the smoking behaviors of caregivers of children with CKD. Material and methods: A cross sectional study was conducted in which caregivers of children and adolescents with CKD were asked to complete a single anonymous self-administered survey. Results: Almost 40% of children and adolescents lived with one or more smokers. Over half of smokers smoked in the presence of their children and in the car. Smokers were significantly less aware of the detrimental effect of SHS exposure on the renal health of their children. Among smokers, almost 70% reported they had not been advised by their child’s nephrologist to quit tobacco use. Conclusion: There is a high prevalence of SHS exposure among children and adolescents with CKD, which may contribute to CVD. Caregivers are not fully aware of the detrimental effects of SHS exposure on the renal health of their children.

## Introduction 

Tobacco use and exposure remain a significant cause of morbidity and mortality, and the Surgeon General of the United States has reported that there is no safe level of exposure to second hand smoke (SHS), which is mostly parental in origin [1]. 

Among children and adolescents with chronic kidney disease (CKD), there is a high prevalence of cardiovascular mortality and morbidity [2]. Risk factors associated with cardiovascular disease (CVD) in these children include hypertension, anemia, and hyperphosphatemia [2]. 

Adult studies have described a clear link between tobacco exposure, onset and progression of CKD, and end stage renal disease [3, 4, 5, 6]. Also, laboratory and clinical based research is highlighting how nicotine and other chemicals in tobacco smoke can directly affect renal tissue in a variety of pathways, resulting in injury [7, 8, 9]. 

Studies have demonstrated a clear relationship between SHS exposure and CVD in adults [10]. In healthy children, exposure to SHS has been associated with elevated blood pressure (BP) [11], endothelial dysfunction [12], dyslipidemia [13], and increased BP load [14]. Furthermore, animal studies have suggested a direct effect of SHS exposure in progression of renal injury [15], raising the possibility that exposure to SHS may affect both cardiovascular and renal outcomes of children with CKD. 

We recently reported a high prevalence of SHS exposure in children and adolescents with CKD [16]. As an initial effort to mitigate exposure, we sought to investigate parental smoking behavior and knowledge regarding SHS exposure in their children with CKD. 

## Methods 

### Study design 

For the purpose of this study, CKD was defined as having: 1) documented primary or secondary glomerulopathy; 2) receiving chronic dialysis; or 3) functioning kidney transplant. A child was classified as exposed to SHS if anyone living in the home was a smoker (cigarettes, cigars, or pipes). 

### Data collection 

Institutional Review Board approval was obtained at each of the six participating centers from the Midwest Pediatric Nephrology Consortium (MWPNC). Surveys were distributed to parents or caregivers who accompanied children with CKD between the ages of 0 and 17 years to the outpatient pediatric nephrology clinic. Caregivers anonymously answered 17 self-administered questions during a routine clinic visit. The questionnaire was answered in private, placed in an envelope, and the envelope sealed by the caregiver. Envelopes were mailed directly to the principal investigator. A maximum of one questionnaire per child was collected. The survey included 4 demographic questions (caregiver’s race and relation to child, and child’s age and type of kidney condition), 9 questions about SHS exposure in the home and family car, 3 questions about the effects of SHS on the child (whether smoking can affect the child’s kidney, cholesterol, or blood pressure), and 1 question about whether the child’s nephrologist had advised the caregiver in the past to stop smoking. 

### Data analysis 

Categorical variables were compared between exposed and non-exposed children using χ^2^-tests. Age of the children, which was non-normally distributed (Kolmogorov-Smirnov test for normality p = 0.003), was compared using a Mann-Whitney test. Nonsmoking and smoking caregivers were compared on questions of the effects of smoking on the child’s kidneys, cholesterol, and blood pressure withχ^2^-tests. Fisher’s exact tests were used to determine whether the number of missing responses differed between the non-smoking and smoking caregivers on these questions. To account for missing data, best/worst case scenario analysis was performed by assigning “no” responses to non-smoking caregivers with missing data, and “yes” responses to smoking caregivers with missing data. For descriptive statistics, frequencies were expressed as the percent of non-missing responses. Age was reported as the median and interquartile range (IQR). Data were analyzed with SAS version 9.2 for Windows^®^ (SAS Institute, Cary, NC, USA). A p-value less than 0.05 was considered statistically significant. 

## Results 

258 eligible caregivers from 6 sites were invited to participate. Three caregivers declined, and 255 completed the survey. Of these, 3 were excluded due to missing data for smoking questions, and 1 was excluded because the child’s age was 20 years. 251 surveys were included in the analyses. The majority of caregivers were the child’s mother (73%), 78% were white, 15% black, and 7% other race. 38% of the children and adolescents had a transplant, 13% were on dialysis, and the rest had a glomerulopathy. The median (IQR) age of the children was 11.0 (8.0) years. 

In 94 of the 251 homes (38%), the child was exposed to SHS from one or more smokers. 69 (28%) of the caregivers smoked, and in 34 (14%) homes, both the caregiver and others living in the home were smokers. Among the 69 caregivers who smoke, 65% of them reported that they smoke within the home. Among maternal caregivers (n = 184), 46 (25%) were smokers. There were no significant differences in demographic characteristics between respondents with SHS exposure compared to respondents without SHS exposure (Table 1). 

In homes with one or more smokers (n – 94), smoking occurred in the presence of the child in 54 of 94 (57%) and in the family car in 54 of 93 non-missing responses (58%). For caregivers who smoked (n = 69), 65% smoked in the presence of the child, and 62% (42 of 68 non-missing responses) smoked in the family car. Rules about smoking differed between homes with no smokers (n = 157) and homes with smokers (n – 94). Smoking was not allowed in 99% of homes with no smoker. This compares to 58 of 94 (62%) homes with one or more smokers where smoking was allowed in the home (p < 0.001). In 27 of 94 (29%) homes with smokers, smoking was allowed in certain rooms only, and in 9% there were no rules. 

A significantly higher proportion of nonsmoking caregivers thought that smoking could affect their child’s kidney, cholesterol, and blood pressure compared to smoking caregivers (Table 2). The groups did not differ in the number of missing responses for any of the three questions, but when best/worst case analysis was performed the proportion of non-smoking caregivers who thought that smoking could affect their child’s cholesterol was not different from the smoking caregivers. For the 69 smokers, 47 (69%) indicated that their child’s nephrologist had not advised them to quit smoking, 21 indicated that they had been advised, and 1 did not respond. 

## Discussion 

Our findings confirm a high prevalence of SHS exposure in children and adolescents with CKD and underscore the potential role exposure may play in this population already at risk for CVD. Among caregivers who smoked, over half reported smoking in the presence of their child, smoking in the home, and in their cars. Compared to non-smokers, a majority of smokers had no rules restricting smoking in the home or car. There were significant differences between smokers and nonsmokers in parental behavior and knowledge regarding the possible effect of SHS exposure on their children. 

Since SHS exposure is strongly associated with CVD in adults [10], it seems reasonable to expect similar effects in children, especially in those who are already at high risk for CVD, such as children with CKD. Indeed, Simonetti et al. [11] demonstrated that elevated BP’s in healthy children were correlated with the number of maternal, but not paternal, cigarettes smoked. A similar finding was described by Oddoze et al. [17]. These studies have added significance among our cohort because most children were accompanied by their mothers, a quarter of whom were smokers. 

In the United States pediatric population, rate of exposure to SHS is estimated at about 8% [18], suggesting our rates of exposure are higher; however, differences in sample size and method of data collection can account for these differences. When compared to rates from self-reported exposure in adolescents with CKD [16], rates are similar, suggesting the robustness of our findings. Compared to other medically at-risk children, rates of exposure in our cohort were slightly lower, with 65% of caregivers who smoked reporting smoking in the home compared to 72% in caregivers of children with cancers [19]. Similar observations were noted with regards to smoking in the family car, a practice that has been found to be a powerful indicator of SHS exposure [20]. In recognition of the impact of exposure within cars, some countries and states within the United States have enacted laws banning smoking in a vehicle with a child as a passenger [21]. 

Our results confirm that caregivers, especially smoking caregivers, are not fully aware of the detrimental health effects exposure to SHS may have on the renal and overall health of their children. Considering that children and adolescents exposed to SHS at home are twice as likely to initiate tobacco use [22], discussion about tobacco cessation with caregivers initiated by the pediatric nephrology team members may be helpful to prevent active tobacco use in these medically at-risk children. Clinicians can take advantage of the chronicity of pediatric CKD care to repeatedly motivate and encourage tobacco intervention strategies in caregivers. The finding among smokers of only 31% being advised by their child’s nephrologist to quit signifies an opportunity to improve counseling rates. Although pediatric nephrologists report several barriers to counseling [23], these barriers can be overcome if concerted efforts are made to address them with existing resources. 

Although the focus of our study was to characterize caregiver behavior and knowledge about smoking, it is important that the clinician is aware of recent research highlighting detrimental effects of tobacco smoke on renal tissue and possible cardiovascular morbidity in children. Recently, nephrotic range proteinuria was independently associated with SHS exposure in a cohort of children with mild to moderate CKD [24]. Higher blood pressures and aortic abnormalities were observed in children exposed to SHS [11, 25]. In adults and animal studies, nicotine has been shown to promote mesangial production [8] and tubulointerstitial fibrosis [15, 26]. Other mechanisms by which SHS may contribute to renal injury include stimulation of proinflammatory cytokines, endothelial dysfunction and intrarenal vasoconstriction [10, 27]. Although we did not investigate a cause and effect relationship between tobacco exposure and renal and cardiac outcomes, based on our findings and recent research, it is prudent that tobacco avoidance counseling be included in the care of a child with CKD whether or not there is a smoker within the family. 

There are several limitations to our study. These include the absence of objective parameters of exposure, such as serum cotinine in children with CKD, the use of a survey instrument that has not been validated, small sample size, and the regional nature of the study. These limitations raise questions about the reliability of exposure history (due to possible recall bias and/or unreliable self-reported data) and how applicable our findings are to the entire pediatric CKD population. Absence of data on socioeconomic status (SES) is another limitation because it is well known that tobacco use is associated with SES. We do not think that the lack of SES data affected our findings because it was not a focus of our study. Others limitations were lack of clinical information like glomerular filtration rate and blood pressure of the children and adolescents with CKD whose caregivers participated in this study. Finally, since the majority of respondents were mothers, it is possible that a selection bias may have affected the results. 

Despite these limitations, we have provided important tobacco exposure data in a population that is high risk for CKD and CVD morbidity and mortality. Larger studies on prevalence and severity of SHS exposure in this population are needed. 

## Acknowledgment 

The study was funded by a grant from the Department of Pediatrics, Wright State University Boonshoft School of Medicine, Dayton, OH, USA. 

The study was presented in part as abstract presentations at the Pediatric Academic Societies (PAS) annual meeting in Boston in May 2012. 

Dr. Laskin is supported by a career development grant in Comparative Effectiveness (1KM1CA15671-01). 

The first author acknowledges assistance of Vida Tyc Ph.D. in conceptualization of the survey document. 

## Conflict of interest 

The authors declare that they have no conflict of interests. 

**Table 1:** Characteristics of the caregivers and children and comparisons of surveys without vs. with SHS exposure.

Characteristic	All surveys No. (%)	Surveys without SHS exposure No. (%)	Surveys with SHS exposure No. (%)	p-value
Caregiver’s relation to child				
Mother	184 (73%)	119 (76%)	65 (69%)	0.16
Father	52 (21%)	32 (20%)	20 (21%)	
Other	15 (6%)	6 (4%)	9 (10%)	
Caregiver’s race (n = 250)				
White	194 (78%)	121 (77%)	73 (78%)	0.91
Black	38 (15%)	23 (15%)	15 (16%)	
Other	18 (7%)	12 (8%)	6 (6%)	
Child’s kidney condition				
Transplant	96 (38%)	69 (44%)	27 (29%)	0.06
Dialysis	33 (13%)	19 (12%)	14 (15%)	
Kidney disease	122 (49%)	69 (44%)	53 (56%)	
Child’s age (years)				
Median (IQR)	11.0 ± 8.0	11.0 ± 8.0	10.0 ± 9.0	0.87
Range	0 – 17	0 – 17	0 – 17	

For all variables, n = 251 unless otherwise indicated.

**Table 2:** Comparison between non-smoking and smoking caregivers on questions regarding whether cigarette smoke can affect child’s kidneys, cholesterol, and blood pressure.

Survey question	Nonsmokers No. (%)	Smokers No. (%)	p-value
Do you think smoking can affect your child’s…?			
Kidneys (n = 241)			
No	16 (9%)	18 (28%)	< 0.001
Yes	160 (91%)	47 (72%)	
Cholesterol (n = 236)			
No	55 (32%)	30 (48%)	0.03
Yes	118 (68%)	33 (52%)	
Blood pressure (n = 245)			
No	14 (8%)	17 (26%)	< 0.001
Yes	165 (92%)	49 (74%)	

p-values for comparisons using best/worst case scenario were 0.007 for kidneys, 0.22 for cholesterol, and 0.002 for blood pressure.
